# Modern Sociology

**Published:** 1901-11-09

**Authors:** 


### Modern Sociology.

### THE RE-APPEARANCE OF FOOT-AND-MOUTH DISEASE.

Last year the authorities of the Board of Agriculture were annoyed to hear that the ailment generally known as "foot-and-mouth disease" had appeared at a farm in Suffolk. For nearly six years the country had been free from this disease among cattle, and, as it is highly contagious, and very difficult to get rid of when once it has appeared, as, moreover, the first step to eliminating it involves the slaughter of all the animals on the farm on which it appears, those who have the agricultural interests of the country at heart were deeply distressed, and the keenest inspection of all animals entering the country has been resorted to to prevent its progress.

The disease, the popular name of which points to the most obvious symptoms, is sometimes called aphthous fever; but this name, in spite of its apparently scientific sound, is by no means accurate, as it suggests that the mouth only is the seat of the disease. The disease is better described as contagious or infectious epizootic eczema, though the vesicles on the tongue and other parts of the mouth form a very prominent symptom, and by causing the affected animal pain and difficulty in eating, are often the first thing to draw attention to the disease. Similar vesicles form above the hoof and between the toes, and cause tenderness in walking. As has been said, the disease is highly infectious, the period of incubation is very short, and all farm animals except horses—cattle, sheep, swine—are subject to it. The ruin which a single case may bring in its train can therefore easily be guessed. Foot-and-mouth disease was first recognised in 1839, and in spite of all efforts to get rid of it, was to be found in the country until 1886. It appeared again in 1892, and again in the latter part of 1894. On each occasion its spread was checked, but again, in January 1900, it made a re-appearance, and cases kept cropping up here and there all through the year, the last case reported being one at Harlow, in Essex, on December 12th.

The highly contagious nature of the disease may be shown by a summary of the first cases reported. The first case appeared on a farm at Fritton, a place in Suffolk, but near the borders of Norfolk, and not far from Yarmouth. The case was reported on January 29, 54 out of 80 cattle, and two out of 40 swine being affected. By the time the veterinary authorities had examined the affected animals the height of the disease was past, and it was thought that more harm might be done by allowing butchers and slaughtermen to approach them than good would be gained by killing them. They were therefore carefully isolated, and when they had recovered their mouths were washed out with a solution of permanganate of potash, and other parts carefully washed with a carbolic solution. No suggestion could be offered as to the source of infection in this case, but as within the first week of February three cases appeared in the neighbourhood of Yarmouth, and two more near Norwich, where the infection seemed to have come with the advent of a bullock brought from one of these places about Yarmouth, it seems likely that Yarmouth was the place whence the disease originally came. The probability is that the infection was carried by imported foreign cattle. It does not follow that there were newly-purchased foreign cattle in any of the places where cases of disease appeared. So infectious is the complaint that if an animal lay for a night in a pen where an infected animal had lain before, it might contract the disease and convey it to other beasts with which it might afterwards associate. Possibly the contagion did not even pass originally directly from one animal to another, but indirectly through the medium of a farmer or cattleman. This possibility of mediate contagion through human beings is one of the great difficulties connected with the stamping out of foot-and-mouth disease. A very notable instance of this was observed during an outbreak in the year 1877. A man whose brother's cattle were affected visited the sick animals out of curiosity, and within a week the disease appeared among his own stock. It becomes the duty therefore of the State veterinary authorities not only to isolate or slaughter all infected animals, but to forbid the movement of animals within the infected area, to look closely after all foreign animals landing at our ports, and to exercise the strictest supervision over the lairs where these animals are penned after their arrival.

How rapidly the disease spreads from one animal to another may be inferred from the statistics of foreign countries where foot-and-mouth disease was prevalent in 1900. In France no fewer than 11,000 premises were said to be infected in October and as many in November. This probably represents about 100,000 cattle suffering from the disease each month. In Belgium reports of its presence came from 12,000 premises, and in Holland there were 24,000 animals reported as affected in November alone. Only the greatest care can keep Britain free from a disease which is so prevalent in neighbouring countries. It is for this reason that the Board of Agriculture hesitates about allowing the importation of store cattle. When foreign animals are slaughtered immediately after their arrival, the possibility of their conveying infection to others is limited, but if they were taken away and mingled with other animals, an epidemic might easily be started which it would be difficult to stamp out.

A very little carelessness in this matter of supervision of imported animals may lead to extremely disastrous results. Thus in Argentina, a country which exports a considerable number of cattle to the English market, foot-and-mouth disease was unknown until 1899. But, with a view to improving their own stock, leading agriculturists in Argentine were in the habit of importing pure bred stock from Europe, and the authorities of the Republic were so confident that their sanitary arrangements were sufficient to prevent diseased cattle entering the country, that they did not hesitate to import animals from countries where this malady was prevalent. Thus it happened that in 1899 the infection came with a cargo of cattle from France. The existence of the disease was not noted until the French cattle had been dispersed over the country and had infected many of the native stock. Even then the Argentine authorities do not seem to have regarded the disease as a serious matter, but they must have been forced to realise the effect it would have on their export cattle trade, when in the second week of April, 1900, the steamer *Ethelhilda* arrived at Deptford with 244 cattle aboard, of which no fewer than 154 were suffering from foot-and-mouth disease. Within six weeks 24 cargoes of diseased animals arrived at Deptford and three at Liverpool. In one instance the effects of the disease had been so fatal on the voyage that of 289 cattle shipped at Buenos Ayres only six were landed alive in this country. In several cases more than half the cargo died on the voyage. An order was immediately issued prohibiting the importation of any more Argentine cattle, an order which will have a serious effect on the agricultural interest in Argentina; but, meanwhile, the British nation is put to considerable anxiety and expense in trying to prevent the infection spreading from these diseased animals to native cattle. The infected Argentines were of course immediately slaughtered, but the sheds and lairs where they had been placed on landing, the drains and all the woodwork about the place had to be carefully cleansed and lime-washed, and the carcases, offal, and manure of the animals disposed of in such a way as to prevent their carrying the infection elsewhere. Elaborate and expensive precautions, it will be seen, but necessary, since, unless the spread of the infection can be stayed, the food supply of the people will suffer.

